# Associations between dementia staging, neuropsychiatric behavioral symptoms, and divorce or separation in late life: A case control study

**DOI:** 10.1371/journal.pone.0289311

**Published:** 2023-08-16

**Authors:** Joan K. Monin, Gail McAvay, Emma Zang, Brent Vander Wyk, Carmen I. Carrión, Heather Allore

**Affiliations:** 1 Social and Behavioral Sciences, Yale School of Public Health, New Haven, CT, United States of America; 2 Internal Medicine, Yale School of Medicine, New Haven, CT, United States of America; 3 Sociology, Yale University, New Haven, CT, United States of America; 4 Neurology, Yale School of Medicine, New Haven, CT, United States of America; 5 Biostatistics, Yale School of Public Health, New Haven, CT, United States of America; University of Missouri Columbia, UNITED STATES

## Abstract

Dementia can be difficult for married couples for many reasons, including the introduction of caregiving burden, loss of intimacy, and financial strain. In this study, we investigated the impact of dementia staging and neuropsychiatric behavioral symptoms on the likelihood of divorce or separation for older adult married couples. For this case-control study, we used data from the National Alzheimer’s Coordinating Center (NACC) Uniform dataset (UDS) versions 2 and 3. This dataset was from 2007 to 2021 and contains standardized clinical information submitted by NIA/NIH Alzheimer’s Disease Research Centers (ADRCs) across the United States (US). This data was from 37 ADRCs. We selected participants who were married or living as married/domestic partners at their initial visit. Cases were defined by a first divorce/separation occurring during the follow-up period, resulting in 291 participants. We selected 5 controls for each married/living as married case and matched on age. Conditional logistic regression estimated the association between overall Neuro Psychiatric Inventory (NPI) score and severity of individual symptoms of the NPI with case/control status, adjusted for education, the CDR® Dementia Staging Instrument score, living situation, symptom informant, sex, and race. Separate analyses were conducted for each symptom. Multiple comparisons were accounted for with the Hochberg method. Later stage of dementia was negatively associated with divorce/separation with an adjusted odds ratio (AOR) = 0.68 (95%CI = 0.50 to 0.93). A higher overall NPI score was positively associated with divorce/separation AOR = 1.08 (95% CI = 1.03 to 1.12,). More severe ratings of agitation/aggression, depression/dysphoria, disinhibition, and elation/euphoria were associated with greater odds of divorce/separation. Among older adults in the US, a later stage of dementia is associated with a lower likelihood of divorce or separation, while having more severe neuropsychiatric behavioral symptoms of agitation/aggression, depression/dysphoria, disinhibition, and elation/euphoria are associated with a higher likelihood of divorce or separation.

## Introduction

Divorce is on the rise for older adult married couples [1]. Yet, little research has examined the antecedents of late life divorce compared to divorce in earlier stages of adult development. It is important to understand what precipitates late life divorce to provide support to older couples who would like to prevent it. Although divorce may be beneficial for some couples and a matter of safety when there is abuse, in general, divorce has substantial negative consequences for the psychological and financial well-being of individuals. This is particularly the case for older adult women [2]. A recent study showed women experienced a 45% decline in their standard of living (measured by an income-to-needs ratio) after divorce. Men on the other hand had a 21% decline in standard of living which persisted over time despite re-partnering. Both women and men experience roughly a 50% drop in wealth from divorce, with re-partnering ameliorative only for women’s wealth. These economic consequences have important implications not only for the couple but for the family and society [3–5]. At the same time, we have seen an increase in dementia prevalence among older adults, with a large literature showing the strain on close relationships that accompanies this disorder [6]. Although there is a very large body of research demonstrating the negative consequences of dementia caregiving for spouses, no studies to our knowledge have examined whether dementia staging and neuropsychiatric behavioral symptoms are associated with divorce or separation for older adult couples.

Previous research has shown that not being married predicts cognitive impairment. For example, the Health and Retirement Study (HRS) (2000–2014) found that older unmarried adults, including cohabiting, divorced/separated, widowed, and never married older adults, were at significantly higher odds of developing greater cognitive impairment, according to the Telephone Interview for Cognitive Status, than their married counterparts [7]. However, no studies have looked at the other direction of this association. Furthermore, previous studies have not examined whether specific types of neuropsychiatric behavioral symptoms are more associated with divorce or separation than others. However, there was a recent study that examined whether health conditions (doctor diagnosed heart disease, cancer, lung disease, or stroke) were related to divorce in a longitudinal analysis using HRS data from 1998 to 2012. That study showed that these chronic conditions were unrelated to the likelihood of divorce after age 50, and this was the case for both husbands and wives’ conditions [8].

Thus, the aim of this study was to examine whether advanced dementia staging and severity of neuropsychiatric behavioral symptoms were associated with the likelihood of divorce or separation in older adulthood. We hypothesized that later stage dementia status would predict a greater likelihood of divorce/separation among older adults. We also hypothesized that the severity of each neuropsychiatric behavioral symptom measured in the Neuro Psychiatric Inventory (NPI) would be associated with a higher likelihood of divorce/separation.

Our first hypothesis is based on past research showing that a later dementia stage is associated with greater psychological [9] and financial [10] burden, as well as relationship dissatisfaction [6] in older adult spouses. Our second hypothesis is based on prior research showing that the NPI symptoms may be even more distressing than the dementia stage itself. One study showed that a spouse’s NPI symptoms were associated with lower marital quality for the caregiving spouse, independent from cognitive status, with apathy having the most distressing effects [11]. In addition to past research, we base our hypotheses on caregiving stress theories which commonly suggest that NPI symptoms lead to vigilance demands to keep the person living with dementia safe and greater perceptions that the person living with dementia is suffering and has lost their sense of self, which in turn can negatively impact the emotional bond of the couple [12, 13]. Further, different symptoms may be difficult for spouses for different reasons. Symptoms such as euphoria, hallucinations, agitation, disinhibition, anxiety, depression, or lack of emotion for apathy may be emotionally distressing. Symptoms, such as lack of appetite, nighttime waking, and motor disturbances may add to burden for spouses because they feel they need to keep the partner safe, taking away time for their own self-care. This study has important implications for society if the likelihood of divorce/separation could be reduced by clinicians aiming their services at relieving the burden of these symptoms on persons living with dementia and their spouses.

## Materials and methods

### The National Alzheimer’s Coordinating Center (NACC) Uniform Data Set (UDS)

The data for this study come from the National Alzheimer’s Coordinating Center (NACC) Uniform Data Set (UDS). This analysis used de-identified data from 37 ADRCs. This database, initiated in 2005, contains standardized clinical information submitted by NIA/NIH Alzheimer’s Disease Research Centers (ADRC) across the United States (US) [14]. Participants are followed annually via in-person clinic, home visits and telephone follow-ups that are ongoing until drop-out or death. All visits were selected as of the December 2021 data freeze. We included Uniform Data Set (UDS) Form Versions 2.0 and 3.0 which were the two major revisions to the database since 2005. Participants were interviewed annually by trained clinicians or clinic personnel, based on information reported by the participant or their co-participant including family members, friends, or clinician. Written informed consent is obtained from all participants and co-participants.

### Participants

**Fig 1:**
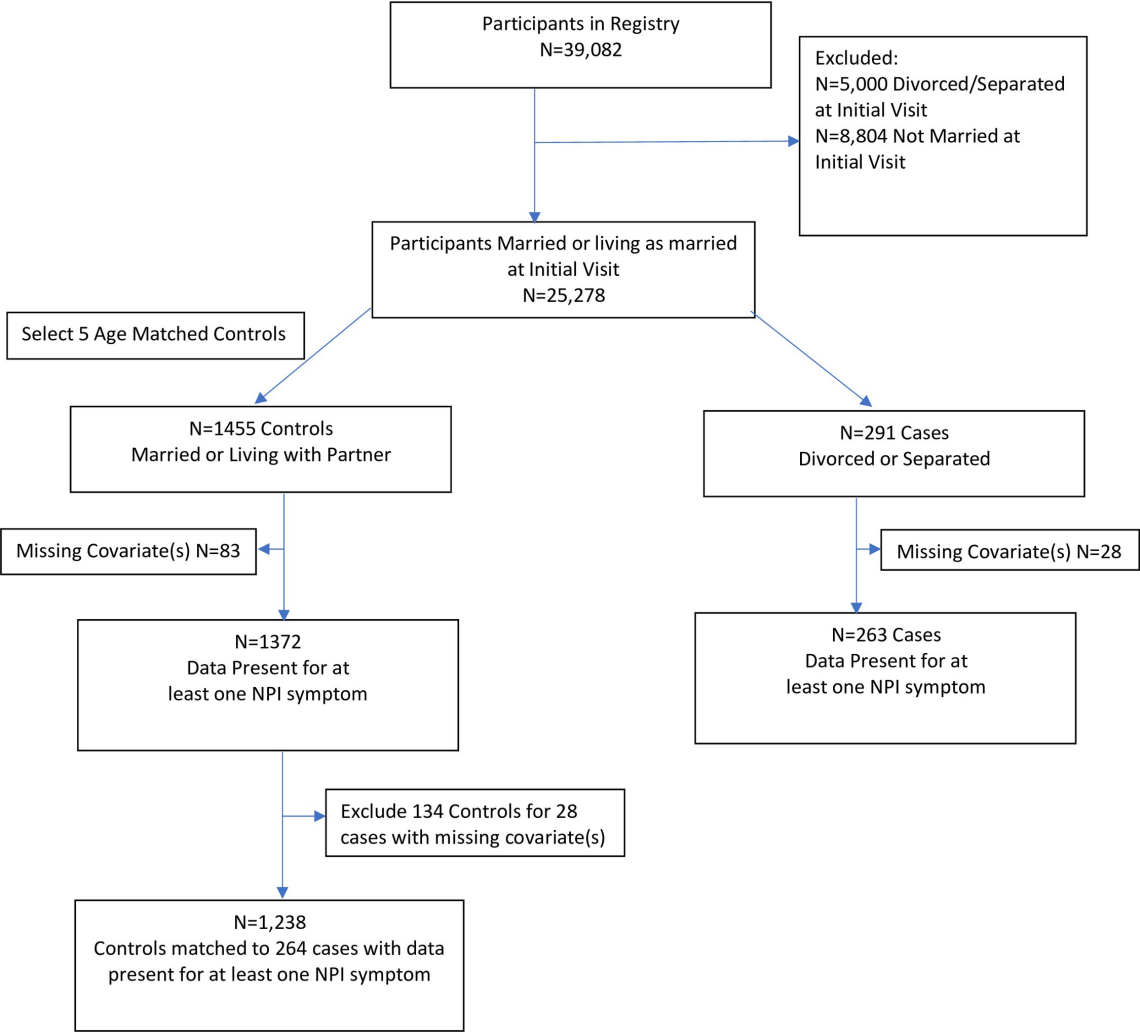
Case-control CONSORT diagram of participants from the National Alzheimer’s Coordinating Center enrolled from 2007 through 2021. The analytic data set included participants who were divorced or separated defined as cases, with five controls selected for each case matched on exact age. Matching was made with replacement, such that 22 participants were matched twice as controls.

We selected participants who were either married or living as married/domestic partners at their initial visit. From this pool, cases for this study were defined by a first divorce or separation occurring during the follow-up period, resulting in 291 participants. We selected 5 controls for each case, who were married or living as married and matched on exact age using a SAS macro developed by Kawabata, Tran and Hines [15]. Matching was made with replacement, such that 22 participants were matched twice as controls. Twenty-eight cases and 217 controls were dropped due to missing data leaving a total of 1501 participants (See Fig 1).

### Divorce or separation

Marital status was assessed at each visit as 1 = Married, 2 = Widowed, 3 = Divorced, 4 = Separated, 5 = Never Married (or marriage was annulled) and 6 = Living as married/domestic partner. Cases were defined as those divorced or separated during follow-up. Controls were those married or living as married.

### Cognitive impairment/dementia staging

The Clinical Dementia Rating (CDR®) Dementia Staging Instrument is a 5-point scale used to characterize six domains of cognitive and functional performance applicable to Alzheimer disease and related dementias: Memory, Orientation, Judgment & Problem Solving, Community Affairs, Home & Hobbies, and Personal Care. The necessary information to make each rating is obtained through a semi-structured interview of the patient and a reliable informant or collateral source (e.g., family member) referred to as the CDR® Assessment Protocol. The core on each item is based on a decline due to cognitive loss, not other impairments such as physical disability. The CDR® global score of function reflects 0.0 = No impairment, 0.5 = Questionable impairment, 1.0 = Mild impairment, 2.0 = Moderate impairment and 3 = Severe impairment [16].

### Neuropsychiatric symptoms

Our primary exposure included 12 symptoms from the NPI [17], that was completed by an informant (spouse, child, or other). Symptoms present in the past month were rated as 0 = No, 1 = Mild, 2 = Moderate and 3 = Severe and included agitation/aggression, anxiety, apathy/indifference, appetite/eating, delusions, depression/dysphoria, disinhibition, elation/euphoria, hallucinations, irritability/lability, motor disturbance and nighttime behaviors. Exposure data and covariates were from the visit prior to the divorce/separation for cases, while control’s exposure data was pulled from the visit prior to the matched visit.

### Covariates

Age in years (33–95), sex (0 = male, 1 = female), years of education (2 to 27), the relationship of the participant and informant (1 = Spouse, 2 = Child, 3 = Other), and whether the informant was living with the participant (1 = Yes, 0 = No) were included as covariates in the analysis. Although we have a representation of multiple races (white, Black/African American, Asian, American Indian/Alaskan, and multiracial) in this sample, the majority of participants was white. Thus, we used the covariate (white = 1, not white = 0). Hispanic/Latino ethnicity (yes = 1, no = 0) was not a significant covariate, and including this variable in the models did not affect the main results.

### Statistical analysis

Descriptive statistics including frequencies, means and standard deviations were calculated for the demographic and clinical variables. Conditional logistic regression was used to estimate the association between the CDR® global score, overall NPI score, both CDR® global score and overall NPI score, and case/control status, adjusted for the previously listed covariates. Separate analyses were conducted for each NPI symptom, and p-values were adjusted for multiple comparisons using the Hochberg method. All analyses were conducted using SAS version 9.4 (SAS Institute Inc., Cary, NC, USA).

## Results and discussion

Sociodemographic and clinical characteristics of the 263 cases and their matched controls are presented in Table 1. Both groups had an average age of 68.9 years due to the matching, while female sex was also equally distributed at 46% in both groups. Cases were less likely to be white than controls, 77.2% and 88.4% respectively, while the mean years of education was approximately 16 in both groups. Most informants were spouses in both groups, but the cases were less likely to have a spouse report on their symptoms than controls, 48.7% versus 86.6%. The informant reports were gathered prior to the divorce/separation. A third of the informants were categorized as another type of relationship in the case group, for example friends/neighbors and siblings, while only 7.3% of informants for controls consisted of other relationships. Finally, 19.0% of cases had child informants as compared to 6.1% for controls, while cases were much less likely to live with their informant, 58.6% than controls 87.7%.

**Table 1:** National Alzheimer’s Coordinating Center (NACC) uniform dataset characteristics of cases and controls.

	Divorced/Separated Cases	Married Age Matched Controls
	(N = 263)	(N = 1238)
Age: Mean (STD)	68.9 (11.2)	68.9 (11.1)
Age Range:	33–95	33–95
Female:	120 (45.6)	566 (45.7)
Race:		
White	203 (77.2)	1094 (88.4)
Black or African American	41 (15.6)	71 (5.7)
American Indian or Alaska Native	0 (0.0)	6 (0.5)
Asian	6 (2.3)	35 (2.8)
Multiracial	13 (4.9)	32 (2.6)
Hispanic/Latino Ethnicity:	22 (8.5)	55 (4.4)
Years of Education: Mean (STD)	15.6 (3.2)	16.0 (2.8)
Informant Relation for NPI:		
Spouse	128 (48.7)	1072 (86.6)
Child	50 (19.0)	75 (6.1)
Other:	85 (32.3)	91 (7.3)
Examples of Other:		
Friend/Neighbor	12 (14.1)	4 (4.4)
Sibling/Sibling in law	4 (4.7)	7 (7.7)
Ex-spouse	3 (3.5)	0 (0.0)
Miscellaneous	9 (10.6)	3 (3.3)
Missing	57 (67.1)	77 (84.6)
Lives with Informant:	154 (58.6)	1086 (87.7)
Clinical Dementia Rating Scale Global Score:		
0·0 = No impairment	140 (53.2)	599 (48.4)
0·5 = Questionable impairment	88 (33.5)	354 (28.6)
1·0 = Mild impairment	21 (8.0)	178 (14.4)
2·0 = Moderate impairment	9 (3.4)	67 (5.4)
3·0 = Severe impairment	5 (1.9)	40 (3.2)

The dementia staging of cases and controls was measured by the CDR® global score, with 53.2% of cases versus 48.4% of controls categorized as having no impairment in functioning, while controls tended to have higher impairment ratings of mild to severe than cases.

The frequencies of the presence of the 12 NPI symptoms in cases and controls are displayed in Table 2, grouped by type of informant and overall. Across informants, the three most frequently reported symptoms for both cases and controls were irritability, anxiety, and depression. Looking at the informant ratings for these symptoms, for both cases and controls adult children reported irritability less frequently, in cases the spouse reported depression most frequently, while among controls both the spouse and adult child reported more depression than the other informants. Over 20% of cases were rated as anxious by all informants, with the spousal informant for controls reporting anxiety most frequently. Hallucinations, elation, and delusions were infrequently reported by informants for both cases and controls, but all three of these symptoms were reported more frequently for cases.

**Table 2:** Presence of Neuropsychiatric Instrument (NPI) symptoms by informant and group.

Symptom:	Informant:	Divorced/Separated Cases (N = 263)		Married Age Matched Controls (N = 1238)	
		n	%	n	%
Agitation/Aggression	Spouse	37	28.9	230	21·5
	Child	8	16·0	11	14·7
	Other	13	15·3	9	9·9
	Total	58	22·0	250	20·2
Anxiety	Spouse	31	25.8	283	27·6
	Child	12	26·7	13	18·6
	Other	16	20·5	15	17·4
	Total	59	24·3	311	26·3
Apathy/Indifference	Spouse	29	22.7	266	24.8
	Child	8	16·0	7	9·3
	Other	15	17·6	6	6·6
	Total	52	19.8	279	22·6
Appetite/Eating	Spouse	20	15·6	170	15.9
	Child	5	10·0	7	9·3
	Other	8	9·5	9	9·9
	Total	33	12.6	189	15·0
Delusions	Spouse	4	3·1	48	4·5
	Child	5	10·0	3	4.0
	Other	5	5·9	1	1·1
	Total	14	5·3	52	4·2
Depression/Dysphoria	Spouse	42	35·0	265	25.9
	Child	11	24·4	19	27.1
	Other	22	27·8	14	16.3
	Total	75	30.7	298	25·3
Disinhibition	Spouse	28	21·9	141	13·2
	Child	4	8·0	7	9·3
	Other	11	12·9	5	5·5
	Total	43	16·3	153	12·4
Elation/Euphoria	Spouse	11	9·2	46	4·5
	Child	2	4·4	0	0
	Other	6	7·6	0	0
	Total	19	7·8	46	3·9
Hallucinations	Spouse	6	4·7	32	3·0
	Child	0	0	1	1·3
	Other	4	4·7	2	2·2
	Total	10	3·8	35	2·8
Irritability/Lability	Spouse	40	31·2	315	29·4
	Child	8	16·0	10	13·3
	Other	26	30·6	19	20·9
	Total	74	28·1	344	27·8
Motor Disturbance	Spouse	14	10.9	132	12·3
	Child	4	8·0	2	2·7
	Other	7	8·2	8	8·8
	Total	25	9·5	142	11·5
Night Behaviors	Spouse	31	24·2	222	20·7
	Child	4	8·0	13	17·6
	Other	19	22·6	8	8·8
	Total	54	20·6	243	19·6

Missing Data in Cases: Anxiety n = 20; Appetite/Eating n = 1; Depression Dysphoria n = 19; Elation/Euphoria n = 19; Night Behaviors n = 1. Missing Data in Controls: Anxiety n = 57; Apathy/Indifference n = 1; Appetite Eating n = 1; Delusions n = 2; Depression Dysphoria n = 59; Disinhibition n = 1; Elation/Euphoria n = 57; Hallucinations n = 1; Irritability/Lability n = 1; Motor Disturbance n = 1; Night Behaviors n = 1.

Our first hypothesis examined whether later dementia stage as measured by the CDR®, was associated with greater likelihood of divorce or separation. As shown in Table 3 under the CDR® Score Alone column, the association between the CDR® score and divorce/separation was not significant, when adjusting for covariates, without the overall NPI score in the model. In contrast, there was a significant positive association between the overall-NPI score and divorce/separation, when adjusting for covariates without CDR® score. Interestingly, when both overall NPI score and CDR® score were included in the same model, the association between dementia stage and divorce/separation became significant, but the direction of the association was not what we had hypothesized. Specifically, an earlier, not later, stage of dementia was associated with divorce/separation when controlling for NPI overall score and the other covariates.

**Table 3:** Conditional logistic regression model results for the association of the Clinical Dementia Rating Score (CDR® global score) and the Neuropsychiatric Inventory Questionnaire (NPI-Q) Score with divorce/separation.

	CDR Score Alone			NPI Score Alone			CDR and NPI Score		
	(N = 1514)			(N = 1317)			(N = 1317)		
	Odds Ratio^*^	95% CI	p-value	Odds Ratio^*^	95% CI	p-value	Odds Ratio^*^	95% CI	p-value
CDR® Global score	0.88	(0.69 to 1.12)	0.2967	··	··	··	0.68	(0.50 to 0.93)	0.0163
NPI Total Score	··	··	··	1.05	(1.01 to 1.09)	0.0149	1.08	(1.03 to 1.12)	0.0007

*Adjusted for education, living with informant, child informant, spousal informant, sex and race. ·· = Not applicable

Our second hypothesis examined the associations between the severity of each NPI-Q symptom and divorce/separation. The results of the 12 conditional logistic regression models are presented in S1–S3 Tables. Among the 12 NPI-Q symptoms, there were significant associations between the severity of agitation/aggression, depression/dysphoria, disinhibition, elation/euphoria, and divorce/separation, with more severe ratings of these symptoms associated with divorce/separation, adjusting for multiple comparisons.

**Fig 2:**
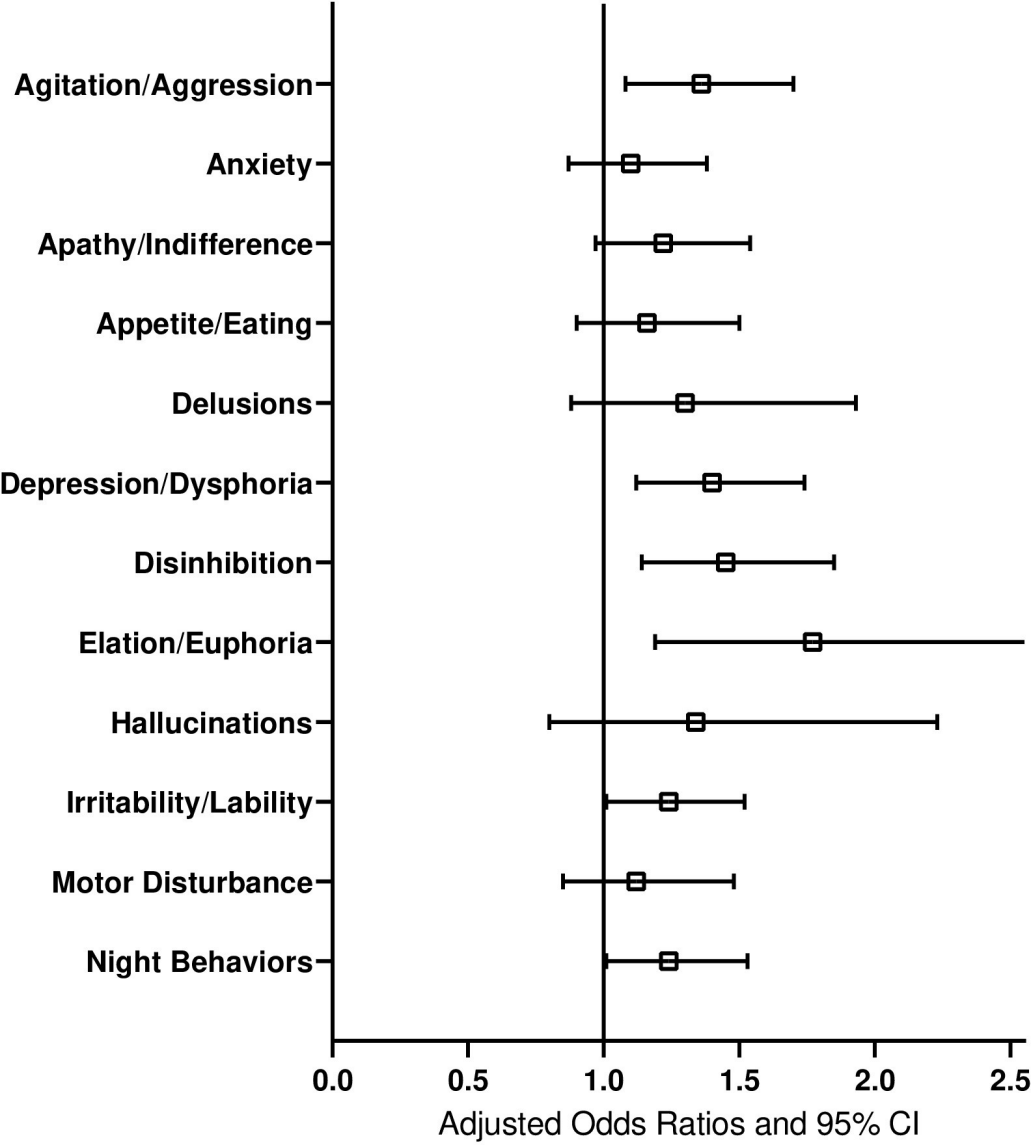
Association between NPI symptom severity and divorce or separation. Note: Results from individual conditional logistic regression models for each symptom. Odds ratios are adjusted for sex, education, race, informant, living with the informant and CDR Global score. CI = Confidence interval; NPI = Neuropsychiatric Instrument.

A graphical presentation of the odds ratios and 95% confidence intervals associated with each symptom are presented in Fig 2. All point estimates were positive, while the lower confidence bound covered the null value for anxiety, apathy/indifference, appetite/eating, delusions, and hallucinations and motor disturbance. Note that these results are not adjusted for multiple comparisons.

In a case control study of older adults in the United States (US), we found that a higher overall NPI score was associated with a greater likelihood of divorce or separation. Further, the following neuropsychiatric behavioral symptom severity were associated with divorce or separation after adjusting for multiple comparisons: agitation/aggression, depression/dysphoria, disinhibition, and elation/euphoria. Divorced/separated cases presented with earlier, not later, stages of dementia, when adjusting for multiple covariates. These findings were reported by different types of informants of the neuropsychiatric behavioral symptoms that are often used in clinical settings, such as spouses, children, and other family members or friends.

Our findings are the first of their kind. They align with other research showing that neuropsychiatric behavioral symptoms are associated with caregiver burden [9] and lower marital satisfaction [11], factors that have tremendous implications for the likelihood of divorce. Other studies have examined whether selected health conditions, not including dementia staging, in older adult couples predict divorce in longitudinal analysis [12]. However, the present study examined dementia staging, a more holistic characterization of dementia status, and neuropsychiatric behavioral symptoms, with more specificity than other studies of the type of health problem and the symptoms that may be burdensome for relationships and with a case control design. Dementia is one of the most burdensome conditions, making it more interpersonally distressing compared to other types of health conditions. Often with conditions that do not include cognitive impairment, the spouse with the condition may maintain the capacity to emotionally support the other spouse for the duration of the chronic or degenerative condition. This is not often the case with dementia.

It is important to note that we did not find support for our hypothesis that later, rather than earlier, stages of dementia would be associated with divorce. Future research should examine the reasons for this. For example, it might be that a spouse of a person who is in the later stages of dementia interprets challenging behaviors as stemming from the disease, whereas spouses with partners who are less impaired may see the behaviors as intentional which may be more damaging to the relationship.

Limitations of this study include that NACC participants are not a statistically based sample of the US population but are referral-based or volunteers. These participants tend to have a higher education and a greater proportion of white race than the US population. Both the sample collection and difference from the general population limits generalizability to the US population [18]. However, there are no studies of this size with such validated longitudinal data to study NPI and dementia staging’s associations with divorce or separation. Moreover, information on the type of informant was collected, and frequencies of the perception of problematic NPI symptoms could be calculated by informant. However, there could be a reporting bias by informants, especially those of cases, as several factors including NPI symptoms could potentiate the dissolution of the marriage. This data was collected over 14 years and there may have been temporal changes over this period. The focus of the ADRCs that contributed data is primarily Alzheimer’s disease and does not measure variables about dyadic processes, such as marital satisfaction. However, the ADRCs collect data on cognitively healthy adults and a variety of associated cognitive disorders, such as vascular dementia, Lewy body dementia, and frontotemporal lobar degeneration. This study did not have sufficient power to study specific cognitive disorders, but our findings show a protective association of worsening dementia staging on divorce or separation. It is also important to acknowledge the possibility that couples dealing with more NPI symptoms are driven to divorce to qualify for benefits including long term care. This does not mean that the relationship has dissolved for the couple, but the divorce is only recognized legally for financial and/or health insurance benefits.

Based on these limitations, we make some suggestions for future studies. First, capturing a more diverse sample in terms of culture and race could help researchers understand multiple mechanisms for the links between dementia stage and symptoms and divorce. For example, cultures differ in terms of their acceptance of divorce [19]. Therefore, couples may be expected to stay together regardless of changes in the well-being of the relationship. Secondly, leveraging longitudinal data to examine changes in living situation during the dementia trajectory could be useful to find earlier warning signs of divorce. Thirdly, it will be important to for future research to examine factors that might mitigate the association between symptom severity and divorce, such as spouses having other people in their network (e.g., an adult child, a professional caregiver) to support them in caregiving.

## Conclusions

These findings have important clinical and societal implications. They suggest that focusing on treatment of symptoms such as agitation/aggression, depression/dysphoria, disinhibition and elation/euphoria in older adults may not only help individuals themselves, but also their spouses, their family, and society. In some healthcare systems, regular psychiatric evaluations and providing appropriate evaluation and treatment are part of the geriatric standard of care; however, many persons living with dementia do not even receive this standard of care. The geriatric mental health workforce is very small despite the growing demands [20]. There are effective behavioral treatments for each of these symptoms in persons living with cognitive impairment [21], and some evidence suggests that interpersonal effects of treatment. For example, a small, randomized control trial showed that treating depression in late life with antidepressants alleviates spousal caregiver burden [22]. Future research should examine the benefits of treating NPI symptoms. Another implication is that the early stages of dementia are sensitive period for older adult couples, where clinicians and social network members may want to provide added relationship support.

## Supporting information

S1 Table Conditional logistic regression models for the association of NPI symptoms of agitation/aggression, anxiety, apathy/indifference and appetite/eating with divorce/separation. (DOCX)

S2 Table Conditional logistic regression models for the association of NPI symptoms of delusions, depression/dysphoria, disinhibition, and elation/euphoria with divorce/separation. (DOCX)

S3 Table Conditional logistic regression models for the association of NPI symptoms of hallucinations, irritability/lability, motor disturbance and night behaviors with divorce/separation. (DOCX)

S1 Dataset (XLS)

S2 Dataset (DOCX)
