# Supplementary material for: Associations between dementia staging, neuropsychiatric behavioral symptoms, and divorce or separation in late life: A case control study
Source: PLoS One. 2023 Aug 16;18(8):e0289311. doi: 10.1371/journal.pone.0289311 (PMC10431668; doi:10.1371/journal.pone.0289311)
Supplement: S1 Table — (DOCX) [file pone.0289311.s001.docx]

| Table S1. Conditional Logistic Regression Models for the Association of NPI symptoms of  Agitation/Aggression, Anxiety, Apathy/Indifference and Appetite/Eating with Divorce/Separation | | | | | | | | | | | | |
| --- | --- | --- | --- | --- | --- | --- | --- | --- | --- | --- | --- | --- |
|  | | | | | | | | | | | | |
| NPI Symptom: | Agitation/ Aggression  (N=1501) | | | Anxiety  (N=1333) | | | Apathy/Indifference  (N=1500) | | | Appetite/Eating  (N=1494) | | |
|  | Odds Ratio | 95% CI | p-value | Odds Ratio | 95% CI | p-value | Odds Ratio | 95% CI | p-value | Odds Ratio | 95% CI | p-value |
| Symptom: | 1.36 | (1.08 to 1.70) | 0.0261 | 1.10 | (0.87 to 1.38) | 0.4344 | 1.22 | (0.97 to 1.54) | 0.1521 | 1.16 | (0.90 to 1.50) | 0.3186 |
| Covariate: |  |  |  |  |  |  |  |  |  |  |  |  |
| Years of education | 0.97 | (0.92 to 1.02) | 0.2333 | 0.96 | (0.91 to 1.02) | 0.1905 | 0.97 | (0.92 to 1.02) | 0.1963 | 0.97 | (0.92 to 1.02) | 0.2423 |
| CRD global score | 0.78 | (0.6 to 1.02) | 0.0677 | 0.84 | (0.64 to 1.1) | 0.1984 | 0.79 | (0.6 to 1.05) | 0.1014 | 0.84 | (0.65 to 1.09) | 0.1923 |
| Lives with informant | 0.75 | (0.47 to 1.19) | 0.2185 | 0.78 | (0.48 to 1.28) | 0.3306 | 0.76 | (0.48 to 1.2) | 0.2403 | 0.75 | (0.47 to 1.19) | 0.2198 |
| Child vs other informant | 0.69 | (0.42 to 1.15) | 0.1542 | 0.64 | (0.37 to 1.09) | 0.1024 | 0.69 | (0.42 to 1.14) | 0.1477 | 0.70 | (0.42 to 1.17) | 0.1722 |
| Female vs male | 0.66 | (0.48 to 0.90) | 0.0091 | 0.68 | (0.49 to 0.94) | 0.0199 | 0.65 | (0.47 to 0.88) | 0.0064 | 0.64 | (0.47 to 0.88) | 0.0056 |
| Spouse vs other informant | 0.14 | (0.08 to 0.23) | 0.0000 | 0.13 | (0.07 to 0.22) | 0.0000 | 0.13 | (0.08 to 0.22) | 0.0000 | 0.14 | (0.08 to 0.23) | 0.0000 |
| White vs not white | 0.53 | (0.36 to 0.79) | 0.0015 | 0.53 | (0.35 to 0.80) | 0.0026 | 0.53 | (0.36 to 0.78) | 0.0013 | 0.53 | (0.36 to 0.78) | 0.0012 |

Note: CI=Confidence interval; p-value for the symptom row adjusted for multiple comparisons.
