# Supplementary material for: Associations between dementia staging, neuropsychiatric behavioral symptoms, and divorce or separation in late life: A case control study
Source: PLoS One. 2023 Aug 16;18(8):e0289311. doi: 10.1371/journal.pone.0289311 (PMC10431668; doi:10.1371/journal.pone.0289311)
Supplement: S2 Table — (DOCX) [file pone.0289311.s002.docx]

| Table S2. Conditional Logistic Regression Models for the Association of NPI symptoms of  Delusions, Depression/Dysphoria, Disinhibition, and Elation/Euphoria with Divorce/Separation | | | | | | | | | | | | |
| --- | --- | --- | --- | --- | --- | --- | --- | --- | --- | --- | --- | --- |
|  | | | | | | | | | | | | |
| NPI Symptom: | Delusions  (N=1499) | | | Depression/Dysphoria  (N=1337) | | | Disinhibition  (N=1500) | | | Elation/Euphoria  (N=1339) | | |
|  | Odds Ratio | 95% CI | p-value | Odds Ratio | 95% CI | p-value | Odds Ratio | 95% CI | p-value | Odds Ratio | 95% CI | p-value |
| Symptom: | 1.30 | (0.88 to 1.93) | 0.2782 | 1.40 | (1.12 to 1.74) | 0.0196 | 1.45 | (1.14 to 1.85) | 0.0196 | 1.77 | (1.19 to 2.63) | 0.0196 |
| Covariate: |  |  |  |  |  |  |  |  |  |  |  |  |
| Years of education | 0.97 | (0.92 to 1.02) | 0.2551 | 0.97 | (0.92 to 1.02) | 0.2576 | 0.97 | (0.92 to 1.02) | 0.1983 | 0.96 | (0.91 to 1.01) | 0.1519 |
| CRD global score | 0.84 | (0.65 to 1.08) | 0.1797 | 0.80 | (0.61 to 1.04) | 0.1020 | 0.78 | (0.6 to 1.01) | 0.0642 | 0.82 | (0.63 to 1.07) | 0.1388 |
| Lives with informant | 0.76 | (0.48 to 1.21) | 0.2436 | 0.72 | (0.43 to 1.18) | 0.1899 | 0.77 | (0.49 to 1.23) | 0.2774 | 0.81 | (0.49 to 1.33) | 0.4011 |
| Child vs other informant | 0.68 | (0.41 to 1.13) | 0.1368 | 0.63 | (0.36 to 1.08) | 0.0929 | 0.70 | (0.42 to 1.17) | 0.1716 | 0.63 | (0.36 to 1.08) | 0.0938 |
| Female vs male | 0.64 | (0.47 to 0.87) | 0.0045 | 0.68 | (0.49 to 0.95) | 0.0218 | 0.66 | (0.48 to 0.90) | 0.0084 | 0.70 | (0.51 to 0.97) | 0.0342 |
| Spouse vs other informant | 0.14 | (0.08 to 0.23) | 0.0000 | 0.13 | (0.07 to 0.23) | 0.0000 | 0.13 | (0.08 to 0.23) | 0.0000 | 0.12 | (0.07 to 0.21) | 0.0000 |
| White vs not white | 0.54 | (0.37 to 0.8) | 0.0019 | 0.53 | (0.35 to 0.8) | 0.0024 | 0.52 | (0.35 to 0.77) | 0.0011 | 0.51 | (0.34 to 0.77) | 0.0013 |

Note: CI=Confidence interval; p-value for the symptom row adjusted for multiple comparisons.
