# Supplementary material for: Associations between dementia staging, neuropsychiatric behavioral symptoms, and divorce or separation in late life: A case control study
Source: PLoS One. 2023 Aug 16;18(8):e0289311. doi: 10.1371/journal.pone.0289311 (PMC10431668; doi:10.1371/journal.pone.0289311)
Supplement: S3 Table — (DOCX) [file pone.0289311.s003.docx]

| Table S3. Conditional Logistic Regression Models for the Association of NPI symptoms of  Hallucinations, Irritability/Lability, Motor Disturbance and Night Behaviors with Divorce/Separation | | | | | | | | | | | | |
| --- | --- | --- | --- | --- | --- | --- | --- | --- | --- | --- | --- | --- |
|  | | | | | | | | | | | | |
| NPI Symptom: | Hallucinations  (N=1500) | | | Irritability/Lability  (N=1500) | | | Motor Disturbance  (N=1500) | | | Night Behaviors  (N=1494) | | |
|  | Odds Ratio | 95% CI | p-value | Odds Ratio | 95% CI | p-value | Odds Ratio | 95% CI | p-value | Odds Ratio | 95% CI | p-value |
| Symptom: | 1.34 | (0.80 to 2.23) | 0.3186 | 1.24 | (1.01 to 1.52) | 0.0892 | 1.12 | (0.85 to 1.48) | 0..4344 | 1.24 | (1.01 to 1.53) | 0.0892 |
| Covariate: |  |  |  |  |  |  |  |  |  |  |  |  |
| Years of education | 0.97 | (0.92 to 1.02) | 0.2047 | 0.97 | (0.92 to 1.02) | 0.2979 | 0.97 | (0.92 to 1.02) | 0.2223 | 0.97 | (0.92 to 1.02) | 0.2427 |
| CRD global score | 0.85 | (0.66 to 1.09) | 0.2080 | 0.82 | (0.63 to 1.06) | 0.1286 | 0.84 | (0.64 to 1.10) | 0.2103 | 0.82 | (0.64 to 1.07) | 0.1408 |
| Lives with informant | 0.76 | (0.48 to 1.2) | 0.2415 | 0.74 | (0.47 to 1.18) | 0.2038 | 0.76 | (0.48 to 1.2) | 0.2386 | 0.75 | (0.47 to 1.19) | 0.2205 |
| Child vs other informant | 0.70 | (0.42 to 1.16) | 0.1691 | 0.71 | (0.43 to 1.18) | 0.1867 | 0.70 | (0.42 to 1.15) | 0.1605 | 0.72 | (0.43 to 1.19) | 0.2016 |
| Female vs male | 0.64 | (0.47 to 0.87) | 0.0049 | 0.66 | (0.48 to 0.9) | 0.0089 | 0.64 | (0.47 to 0.87) | 0.0050 | 0.65 | (0.48 to 0.89) | 0.0075 |
| Spouse vs other informant | 0.14 | (0.08 to 0.23) | 0.0000 | 0.14 | (0.08 to 0.23) | 0.0000 | 0.14 | (0.08 to 0.23) | 0.0000 | 0.14 | (0.08 to 0.23) | 0.0000 |
| White vs not white | 0.53 | (0.36 to 0.78) | 0.0012 | 0.53 | (0.36 to 0.78) | 0.0013 | 0.53 | (0.36 to 0.78) | 0.0013 | 0.52 | (0.36 to 0.77) | 0.0010 |

Note: CI=Confidence interval; p-value for the symptom row adjusted for multiple comparisons.
