# Supplementary material for: Associations between dementia staging, neuropsychiatric behavioral symptoms, and divorce or separation in late life: A case control study
Source: PLoS One. 2023 Aug 16;18(8):e0289311. doi: 10.1371/journal.pone.0289311 (PMC10431668; doi:10.1371/journal.pone.0289311)
Supplement: S2 Dataset — (DOCX) [file pone.0289311.s005.docx]

Supplement 4 Codebook for Excel Database

Excel sheets consist of the data for producing all tables. Blank cells reflect missing data.

| Codebook for Table 1: Sheet named Table1 | | |
| --- | --- | --- |
| Variable | Label | Codes |
| NACCID | NACC ID | - |
| CASE_ID | ID for Case | - |
| DIVSEP | Case Control Status | 1=Case; 0=Control |
| AGE | Age in Years | 33-95 |
| FEMALE | Female Sex | 1=Yes; 0=No |
| L_NACCIHR | Race | 1=White; 2=Black or African American; 3=American Indian or Alaska Native; 5=Asian, 6=Multiracial |
| HISPANIC | Hispanic/Latino Ethnicity | 1=Yes; 0=No; |
| EDUC | Years of Education | 2-27 |
| L_NPIQINF | Informant Relation for NPI | 1=Spouse; 2=Child; 3=Other |
| INFGRP | Examples of Other Type of Informant | Character Data Coded:  Friend/Neighbor; Sibling/Sibling in law);  Ex Spouse/Partner; Miscellaneous; Not Applicable |
| L_INLIVWTH | Lives with Informant | 1=Yes; 0=No |
| L_CDRGLOB | Clinical Dementia Rating Scale Score | 0=No impairment; 0.5=Questionable impairment; 1=Mild impairment; 2=Moderate impairment; 3=Severe impairment |

| Codebook for Table 2: Sheet named Table2 | | |
| --- | --- | --- |
| Variable | Label | Codes |
| NACCID | NACC ID | - |
| CASE_ID | ID for Case | - |
| DIVSEP | Case Control Status | 1=Case; 0=Control |
| L_NPIQINF | Informant Relation for NPI | 1=Spouse; 2=Child; 3=Other |
| L_AGITSEV | NPI Agitation/Aggression | 1=Yes; 0=No |
| L_ANXSEV | NPI Anxiety | 1=Yes; 0=No |
| L_APASEV | Apathy/Indifference | 1=Yes; 0=No |
| L_APPSEV | NPI Appetite | 1=Yes; 0=No |
| L_DELSEV | NPI Delusions | 1=Yes; 0=No |
| L_DEPSEV | NPI Depression/Dysphoria | 1=Yes; 0=No |
| L_DISNSEV | NPI Disinhibition | 1=Yes; 0=No |
| L_ELATSEV | NPI Elation/Euphoria | 1=Yes; 0=No |
| L_HALLSEV | NPI Hallucinations | 1=Yes; 0=No |
| L_IRRSEV | NPI Irritability/Lability | 1=Yes; 0=No |
| L_MOTSEV | NPI Motor Disturbance | 1=Yes; 0=No |
| L_NITESEV | NPI Night Behaviors | 1=Yes; 0=No |

| Codebook for Table 3 CDR Alone Column: Sheet named Table3_Cdralone | | |
| --- | --- | --- |
| Variable | Label | Codes |
| NACCID | NACC ID | - |
| CASE_ID | ID for Case | - |
| DIVSEP | Case Control Status | 1=Case; 0=Control |
| EDUC | Year Education | 2-27 |
| L_CDRGLOB | Clinical Dementia Rating Scale Score | 0=No impairment; 0.5=Questionable impairment; 1=Mild impairment; 2=Moderate impairment; 3=Severe impairment |
| L_INLIVWTH | Living with Informant | 1=Yes; 0=No |
| FEMALE | SEX | 1=Female; 0=Male |
| WHITE | White Race | 1=Yes; 0=No |
| SPOUSE | Spousal Informant | 1=Yes; 0=No |
| CHILD | Child Informant | 1=Yes; 0=No |

| Codebook for Table 3 NPI Alone Column: Sheet named Table3_Npialone | | |
| --- | --- | --- |
| Variable | Label | Codes |
| NACCID | NACC ID | - |
| CASE_ID | ID for Case | - |
| DIVSEP | Case Control Status | 1=Case; 0=Control |
| EDUC | Year Education | 2-27 |
| L_TOTNPI | Total NPI Score | 0-22 |
| L_INLIVWTH | Living with Informant | 1=Yes; 0=No |
| FEMALE | SEX | 1=Female; 0=Male |
| WHITE | White Race | 1=Yes; 0=No |
| SPOUSE | Spousal Informant | 1=Yes; 0=No |
| CHILD | Child Informant | 1=Yes; 0=No |

| Codebook for Table 3 CDR and NPI Column: Sheet named Table3_Cdr_Npi | | |
| --- | --- | --- |
| Variable | Label | Codes |
| NACCID | NACC ID | - |
| CASE_ID | ID for Case | - |
| DIVSEP | Case Control Status | 1=Case; 0=Control |
| EDUC | Year Education | 2-27 |
| L_CDRGLOB | Clinical Dementia Rating Scale Score | 0=No impairment; 0.5=Questionable impairment; 1=Mild impairment; 2=Moderate impairment; 3=Severe impairment |
| L_INLIVWTH | Living with Informant | 1=Yes; 0=No |
| FEMALE | SEX | 1=Female; 0=Male |
| WHITE | White Race | 1=Yes; 0=No |
| SPOUSE | Spousal Informant | 1=Yes; 0=No |
| CHILD | Child Informant | 1=Yes; 0=No |
| L_TOTNPI | Total NPI Score | 0-22 |

| Codebook for Table S1 Agitation Model: Sheet named Model_Agitsev | | |
| --- | --- | --- |
| Variable | Label | Codes |
| NACCID | NACC ID | - |
| CASE_ID | ID for Case | - |
| DIVSEP | Case Control Status | 1=Case; 0=Control |
| EDUC | Year Education | 2-27 |
| L_AGITSEV | NPI Agitation/Aggression | 0-3 |
| L_CDRGLOB | Clinical Dementia Rating Scale Score | 0=No impairment; 0.5=Questionable impairment; 1=Mild impairment; 2=Moderate impairment; 3=Severe impairment |
| L_INLIVWTH | Living with Informant | 1=Yes; 0=No |
| FEMALE | SEX | 1=Female; 0=Male |
| WHITE | White Race | 1=Yes; 0=No |
| SPOUSE | Spousal Informant | 1=Yes; 0=No |
| CHILD | Child Informant | 1=Yes; 0=No |

| Codebook for Table S1 Anxiety Model: Sheet named Model_Anxsev | | |
| --- | --- | --- |
| Variable | Label | Codes |
| NACCID | NACC ID | - |
| CASE_ID | ID for Case | - |
| DIVSEP | Case Control Status | 1=Case; 0=Control |
| EDUC | Year Education | 2-27 |
| L_ANXSEV | NPI Anxiety | 0-3 |
| L_CDRGLOB | Clinical Dementia Rating Scale Score | 0=No impairment; 0.5=Questionable impairment; 1=Mild impairment; 2=Moderate impairment; 3=Severe impairment |
| L_INLIVWTH | Living with Informant | 1=Yes; 0=No |
| FEMALE | SEX | 1=Female; 0=Male |
| WHITE | White Race | 1=Yes; 0=No |
| SPOUSE | Spousal Informant | 1=Yes; 0=No |
| CHILD | Child Informant | 1=Yes; 0=No |

| Codebook for Table S1 Apathy Model: Sheet named Model_Apasev | | |
| --- | --- | --- |
| Variable | Label | Codes |
| NACCID | NACC ID | - |
| CASE_ID | ID for Case | - |
| DIVSEP | Case Control Status | 1=Case; 0=Control |
| EDUC | Year Education | 2-27 |
| L_APASEV | NPI Apathy/Indifference | 0-3 |
| L_CDRGLOB | Clinical Dementia Rating Scale Score | 0=No impairment; 0.5=Questionable impairment; 1=Mild impairment; 2=Moderate impairment; 3=Severe impairment |
| L_INLIVWTH | Living with Informant | 1=Yes; 0=No |
| FEMALE | SEX | 1=Female; 0=Male |
| WHITE | White Race | 1=Yes; 0=No |
| SPOUSE | Spousal Informant | 1=Yes; 0=No |
| CHILD | Child Informant | 1=Yes; 0=No |

| Codebook for Table S1 Appetite Model: Sheet named Model_Appsev | | |
| --- | --- | --- |
| Variable | Label | Codes |
| NACCID | NACC ID | - |
| CASE_ID | ID for Case | - |
| DIVSEP | Case Control Status | 1=Case; 0=Control |
| EDUC | Year Education | 2-27 |
| L_APPSEV | NPI Appetite/Eating | 0-3 |
| L_CDRGLOB | Clinical Dementia Rating Scale Score | 0=No impairment; 0.5=Questionable impairment; 1=Mild impairment; 2=Moderate impairment; 3=Severe impairment |
| L_INLIVWTH | Living with Informant | 1=Yes; 0=No |
| FEMALE | SEX | 1=Female; 0=Male |
| WHITE | White Race | 1=Yes; 0=No |
| SPOUSE | Spousal Informant | 1=Yes; 0=No |
| CHILD | Child Informant | 1=Yes; 0=No |

| Codebook for Table S2 Delusions Model: Sheet named Model_Delsev | | |
| --- | --- | --- |
| Variable | Label | Codes |
| NACCID | NACC ID | - |
| CASE_ID | ID for Case | - |
| DIVSEP | Case Control Status | 1=Case; 0=Control |
| EDUC | Year Education | 2-27 |
| L_DELSEV | NPI Delusions | 0-3 |
| L_CDRGLOB | Clinical Dementia Rating Scale Score | 0=No impairment; 0.5=Questionable impairment; 1=Mild impairment; 2=Moderate impairment; 3=Severe impairment |
| L_INLIVWTH | Living with Informant | 1=Yes; 0=No |
| FEMALE | SEX | 1=Female; 0=Male |
| WHITE | White Race | 1=Yes; 0=No |
| SPOUSE | Spousal Informant | 1=Yes; 0=No |
| CHILD | Child Informant | 1=Yes; 0=No |

| Codebook for Table S2 Depression Model: Sheet named Model_Depsev | | |
| --- | --- | --- |
| Variable | Label | Codes |
| NACCID | NACC ID | - |
| CASE_ID | ID for Case | - |
| DIVSEP | Case Control Status | 1=Case; 0=Control |
| EDUC | Year Education | 2-27 |
| L_DEPDSEV | NPI Depression/Dysphoria | 0-3 |
| L_CDRGLOB | Clinical Dementia Rating Scale Score | 0=No impairment; 0.5=Questionable impairment; 1=Mild impairment; 2=Moderate impairment; 3=Severe impairment |
| L_INLIVWTH | Living with Informant | 1=Yes; 0=No |
| FEMALE | SEX | 1=Female; 0=Male |
| WHITE | White Race | 1=Yes; 0=No |
| SPOUSE | Spousal Informant | 1=Yes; 0=No |
| CHILD | Child Informant | 1=Yes; 0=No |

| Codebook for Table S2 Disinhibition Model: Sheet named Model_Disnsev | | |
| --- | --- | --- |
| Variable | Label | Codes |
| NACCID | NACC ID | - |
| CASE_ID | ID for Case | - |
| DIVSEP | Case Control Status | 1=Case; 0=Control |
| EDUC | Year Education | 2-27 |
| L_DISNSEV | NPI Disinhibition | 0-3 |
| L_CDRGLOB | Clinical Dementia Rating Scale Score | 0=No impairment; 0.5=Questionable impairment; 1=Mild impairment; 2=Moderate impairment; 3=Severe impairment |
| L_INLIVWTH | Living with Informant | 1=Yes; 0=No |
| FEMALE | SEX | 1=Female; 0=Male |
| WHITE | White Race | 1=Yes; 0=No |
| SPOUSE | Spousal Informant | 1=Yes; 0=No |
| CHILD | Child Informant | 1=Yes; 0=No |

| Codebook for Table S2 Elation Model: Sheet named Model_Elatsev | | |
| --- | --- | --- |
| Variable | Label | Codes |
| NACCID | NACC ID | - |
| CASE_ID | ID for Case | - |
| DIVSEP | Case Control Status | 1=Case; 0=Control |
| EDUC | Year Education | 2-27 |
| L_ELATSEV | NPI Elation/Euphoria | 0-3 |
| L_CDRGLOB | Clinical Dementia Rating Scale Score | 0=No impairment; 0.5=Questionable impairment; 1=Mild impairment; 2=Moderate impairment; 3=Severe impairment |
| L_INLIVWTH | Living with Informant | 1=Yes; 0=No |
| FEMALE | SEX | 1=Female; 0=Male |
| WHITE | White Race | 1=Yes; 0=No |
| SPOUSE | Spousal Informant | 1=Yes; 0=No |
| CHILD | Child Informant | 1=Yes; 0=No |

| Codebook for Table S3 Hallucinations Model: Sheet named Model_Hallsev | | |
| --- | --- | --- |
| Variable | Label | Codes |
| NACCID | NACC ID | - |
| CASE_ID | ID for Case | - |
| DIVSEP | Case Control Status | 1=Case; 0=Control |
| EDUC | Year Education | 2-27 |
| L_HALLSEV | NPI Hallucinations | 0-3 |
| L_CDRGLOB | Clinical Dementia Rating Scale Score | 0=No impairment; 0.5=Questionable impairment; 1=Mild impairment; 2=Moderate impairment; 3=Severe impairment |
| L_INLIVWTH | Living with Informant | 1=Yes; 0=No |
| FEMALE | SEX | 1=Female; 0=Male |
| WHITE | White Race | 1=Yes; 0=No |
| SPOUSE | Spousal Informant | 1=Yes; 0=No |
| CHILD | Child Informant | 1=Yes; 0=No |

| Codebook for Table S3 Irritability Model: Sheet named Model_Irrsev | | |
| --- | --- | --- |
| Variable | Label | Codes |
| NACCID | NACC ID | - |
| CASE_ID | ID for Case | - |
| DIVSEP | Case Control Status | 1=Case; 0=Control |
| EDUC | Year Education | 2-27 |
| L_IRRSEV | NPI Irritability/Lability | 0-3 |
| L_CDRGLOB | Clinical Dementia Rating Scale Score | 0=No impairment; 0.5=Questionable impairment; 1=Mild impairment; 2=Moderate impairment; 3=Severe impairment |
| L_INLIVWTH | Living with Informant | 1=Yes; 0=No |
| FEMALE | SEX | 1=Female; 0=Male |
| WHITE | White Race | 1=Yes; 0=No |
| SPOUSE | Spousal Informant | 1=Yes; 0=No |
| CHILD | Child Informant | 1=Yes; 0=No |

| Codebook for Table S3 Motor Disturbance Model: Sheet named Model Motsev | | |
| --- | --- | --- |
| Variable | Label | Codes |
| NACCID | NACC ID | - |
| CASE_ID | ID for Case | - |
| DIVSEP | Case Control Status | 1=Case; 0=Control |
| EDUC | Year Education | 2-27 |
| L_MOTSEV | NPI Motor Disturbance | 0-3 |
| L_CDRGLOB | Clinical Dementia Rating Scale Score | 0=No impairment; 0.5=Questionable impairment; 1=Mild impairment; 2=Moderate impairment; 3=Severe impairment |
| L_INLIVWTH | Living with Informant | 1=Yes; 0=No |
| FEMALE | SEX | 1=Female; 0=Male |
| WHITE | White Race | 1=Yes; 0=No |
| SPOUSE | Spousal Informant | 1=Yes; 0=No |
| CHILD | Child Informant | 1=Yes; 0=No |

| Codebook for Table S3 Night Behaviors: Sheet named Model_Nitesev | | |
| --- | --- | --- |
| Variable | Label | Codes |
| NACCID | NACC ID | - |
| CASE_ID | ID for Case | - |
| DIVSEP | Case Control Status | 1=Case; 0=Control |
| EDUC | Year Education | 2-27 |
| L_NITESEV | NPI Night Behaviors | 0-3 |
| L_CDRGLOB | Clinical Dementia Rating Scale Score | 0=No impairment; 0.5=Questionable impairment; 1=Mild impairment; 2=Moderate impairment; 3=Severe impairment |
| L_INLIVWTH | Living with Informant | 1=Yes; 0=No |
| FEMALE | SEX | 1=Female; 0=Male |
| WHITE | White Race | 1=Yes; 0=No |
| SPOUSE | Spousal Informant | 1=Yes; 0=No |
| CHILD | Child Informant | 1=Yes; 0=No |
